# Supplementary figures and images for: Ribosome subunit attrition and activation of the p53–MDM4 axis dominate the response of MLL-rearranged cancer cells to WDR5 WIN site inhibition
Source: eLife. 2024 Apr 29;12:RP90683. doi: 10.7554/eLife.90683 (PMC11057873; doi:10.7554/eLife.90683)

Figure 3—source data 3

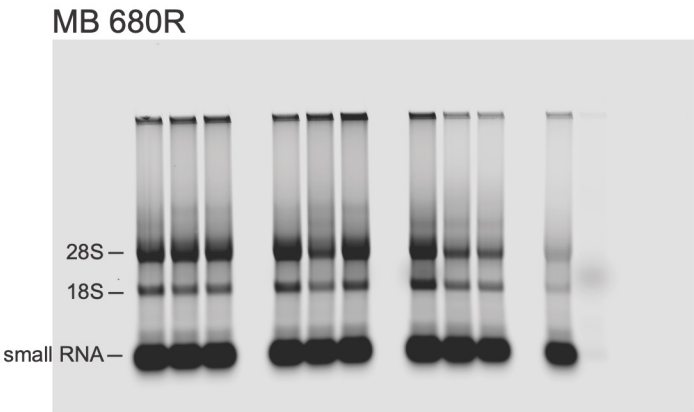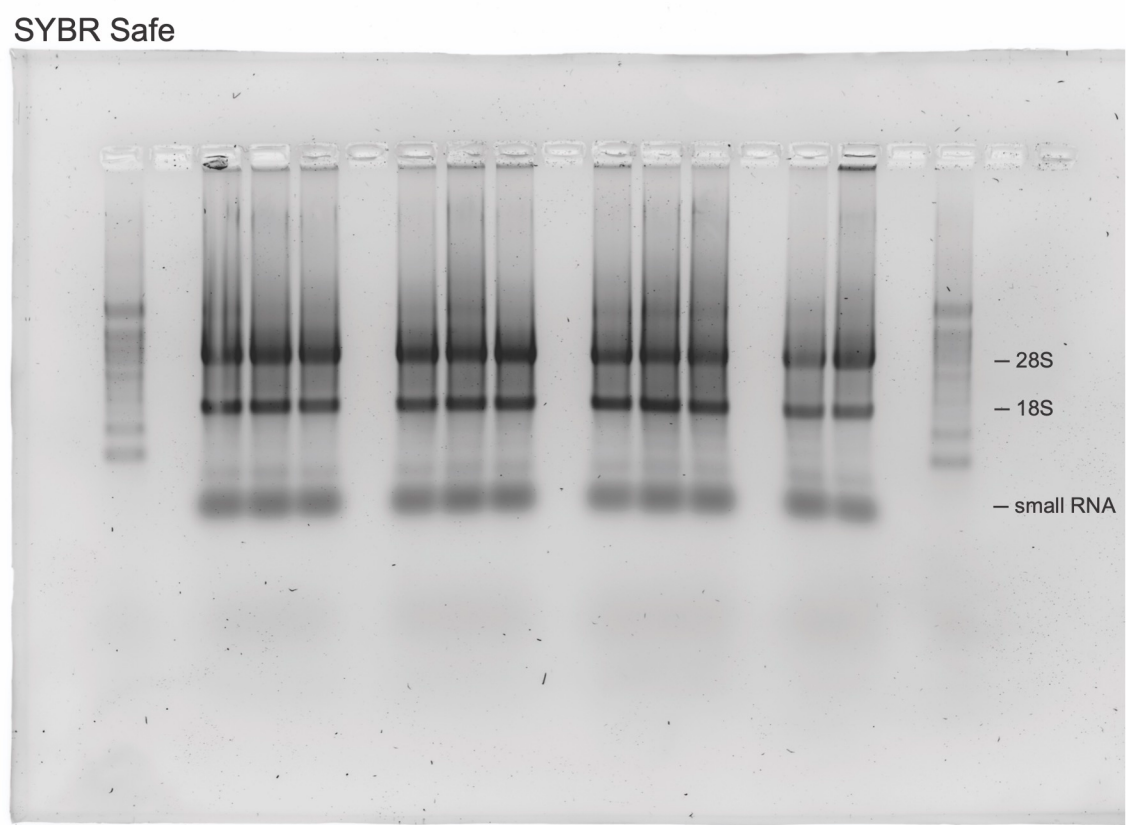

Supplement: Figure 3—figure supplement 3—source data 1. [file elife-90683-fig3-figsupp3-data1.pdf]

Figure 6—source data 1

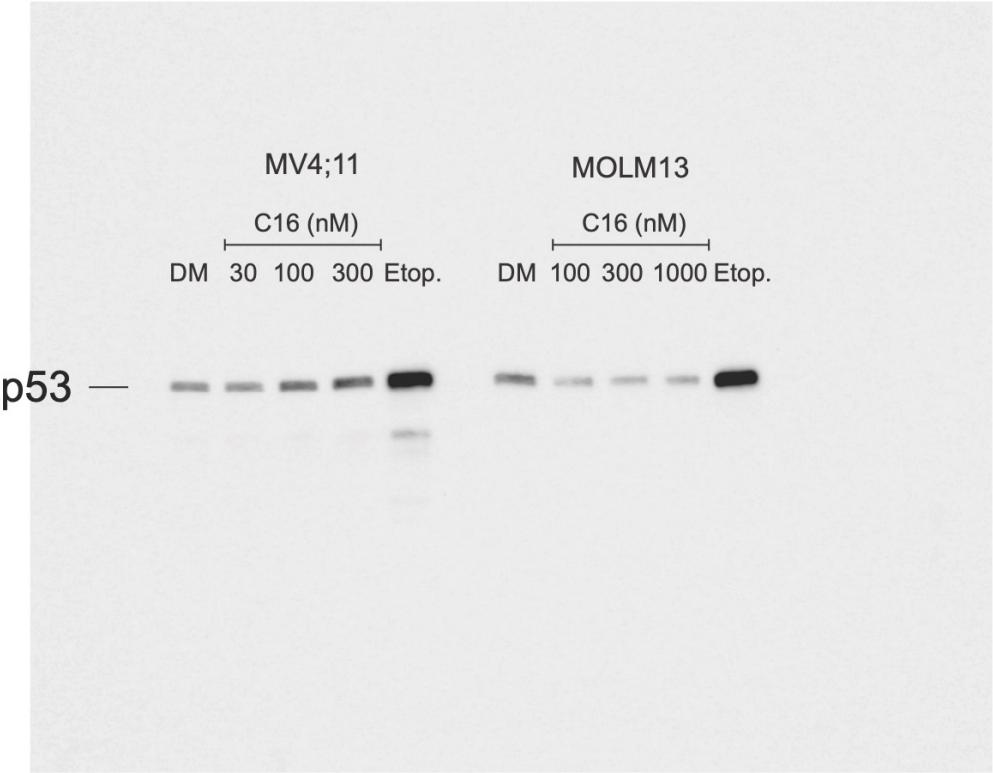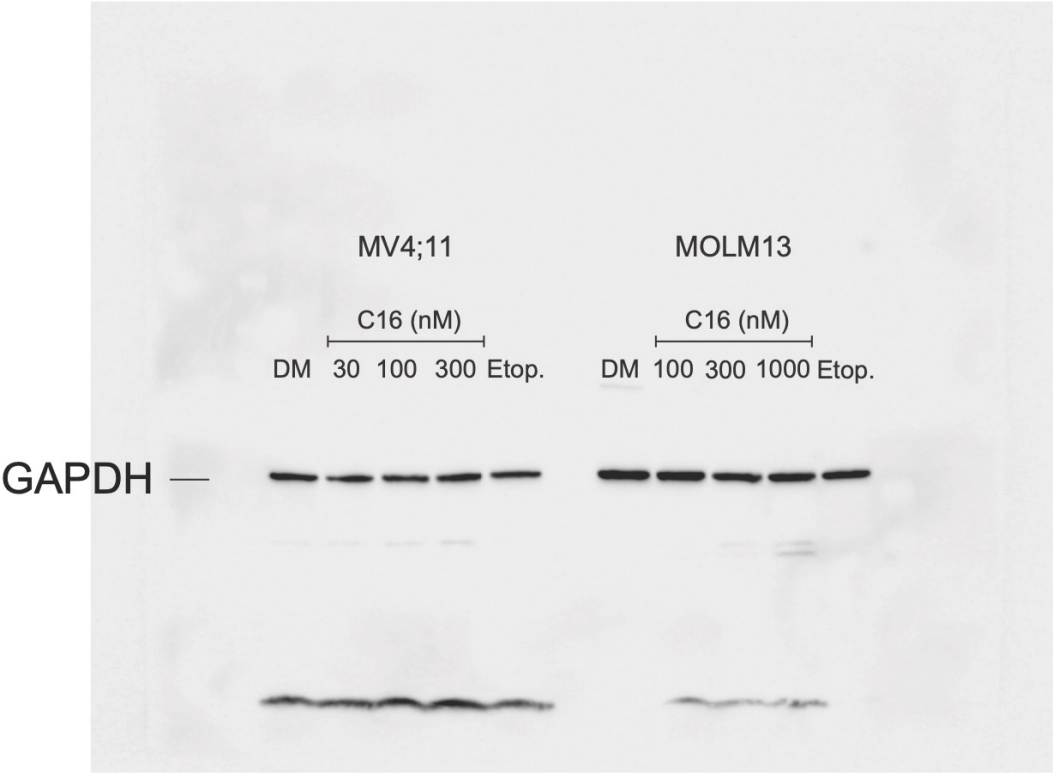

Supplement: Figure 6—source data 1. [file elife-90683-fig6-data1.pdf]

Figure 6—source data 3

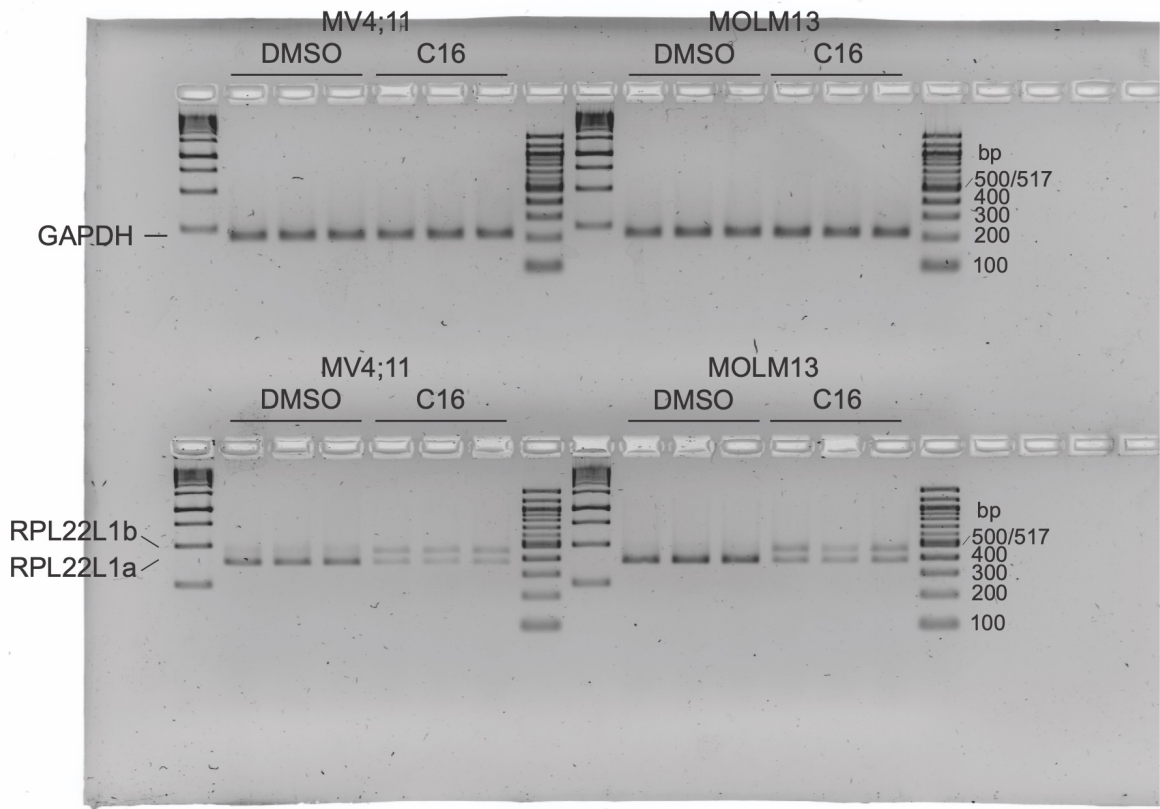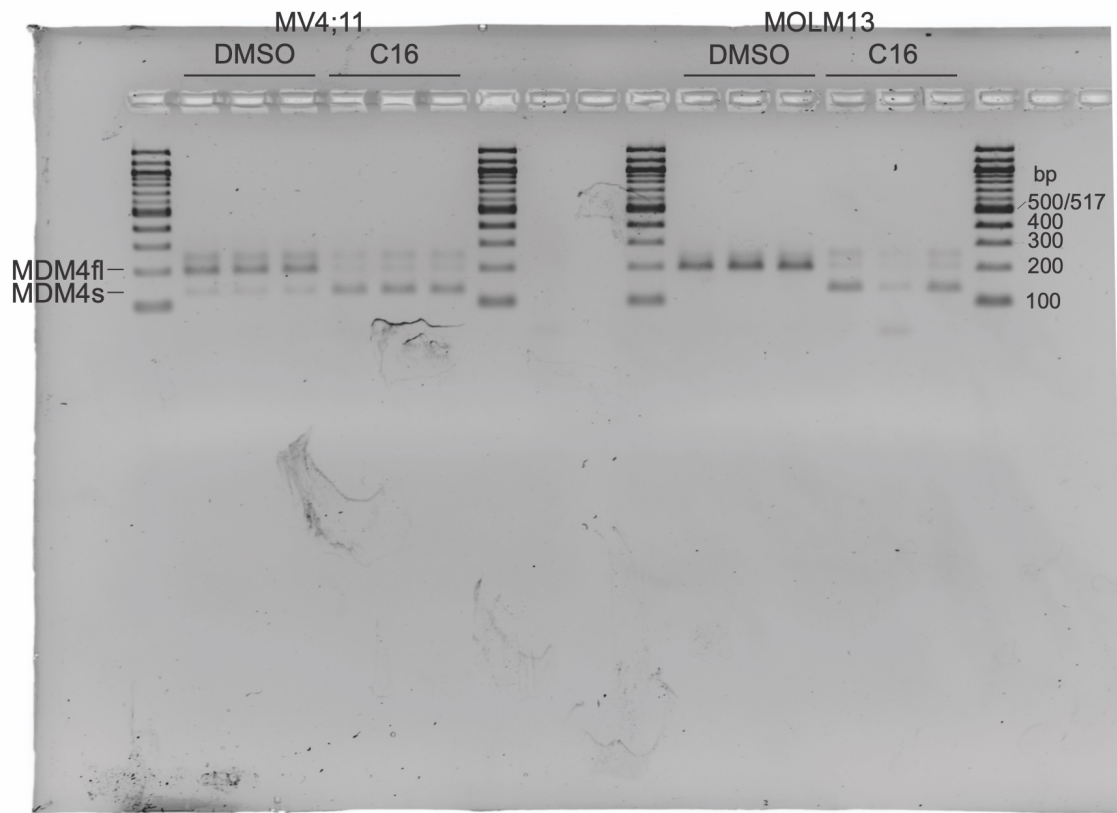

Supplement: Figure 6—source data 3. [file elife-90683-fig6-data3.pdf]

**Figure 6—source data 4**

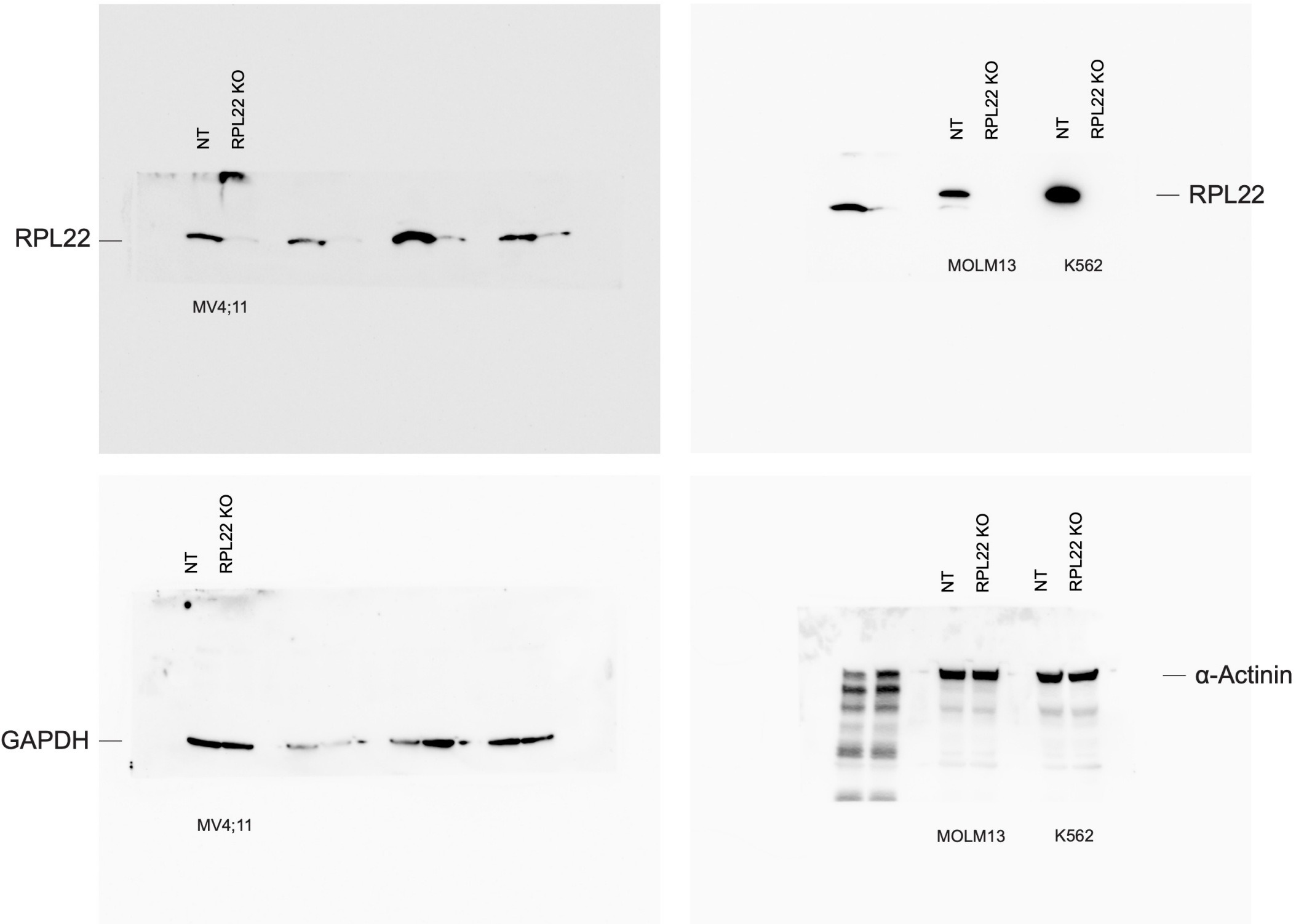

Supplement: Figure 6—source data 4. [file elife-90683-fig6-data4.pdf]

Figure 6—source data 9

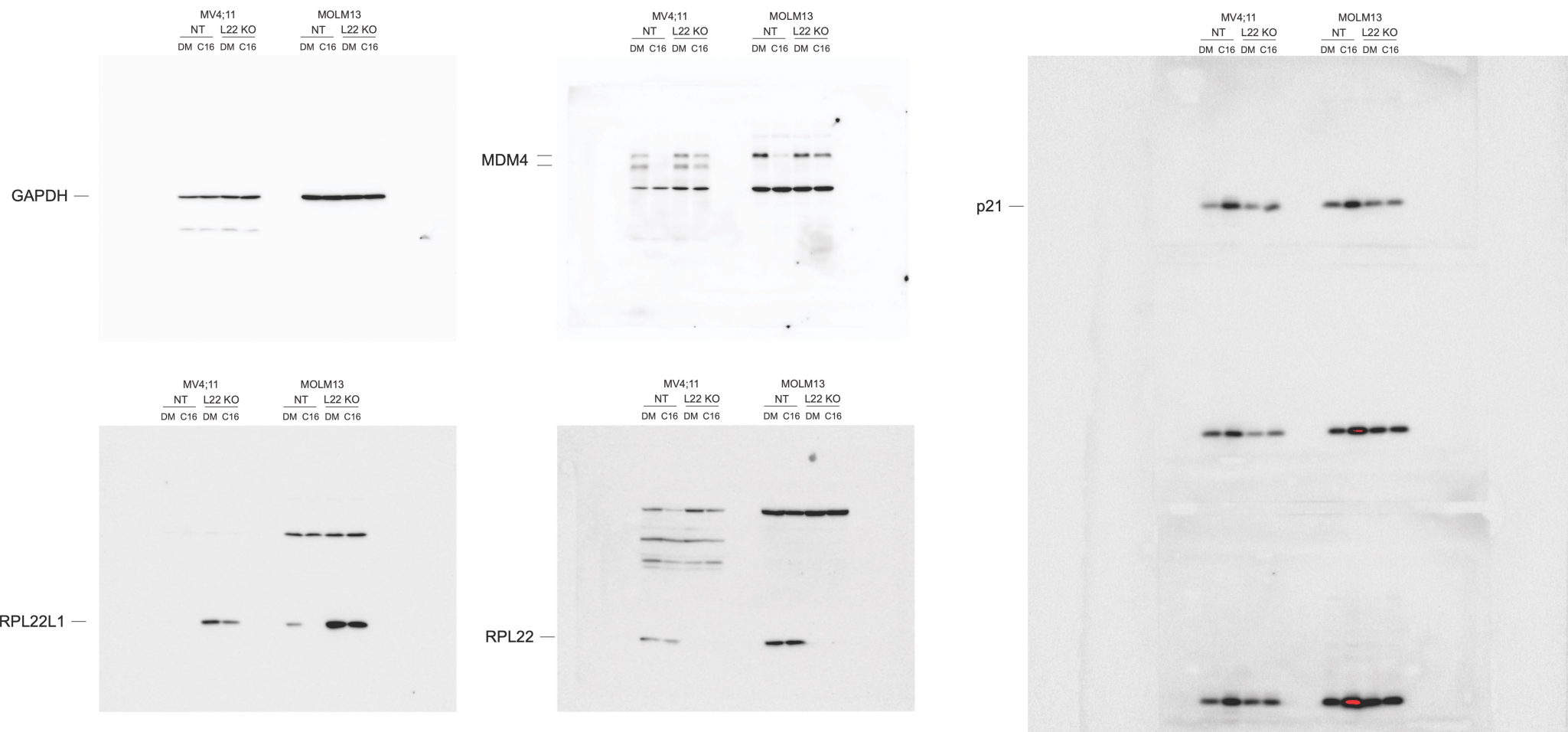

Supplement: Figure 6—source data 9. [file elife-90683-fig6-data9.pdf]
