# Supplementary material for: Ribosome subunit attrition and activation of the p53–MDM4 axis dominate the response of MLL-rearranged cancer cells to WDR5 WIN site inhibition
Source: eLife. 2024 Apr 29;12:RP90683. doi: 10.7554/eLife.90683 (PMC11057873; doi:10.7554/eLife.90683)

Figure 6–source data 5

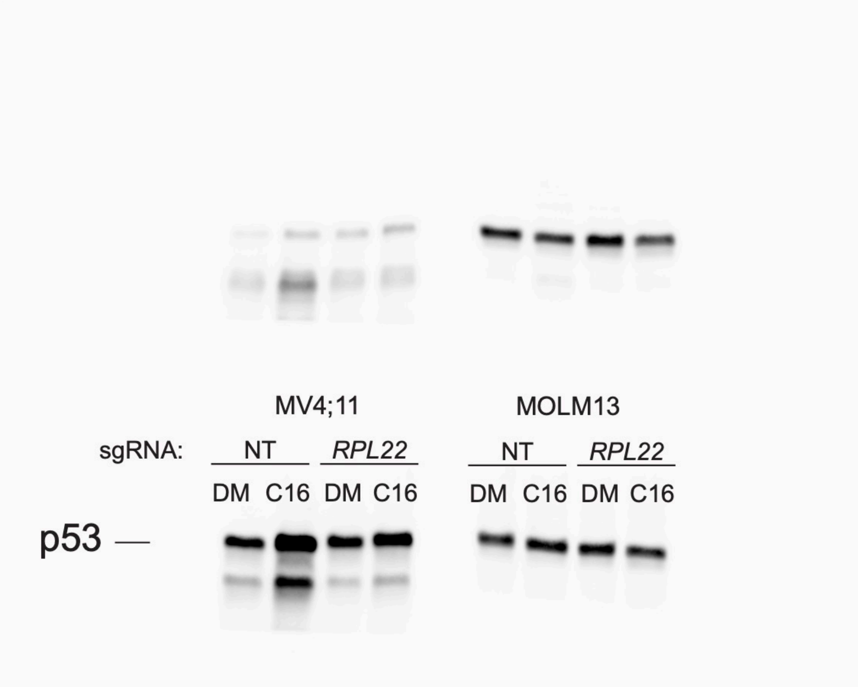

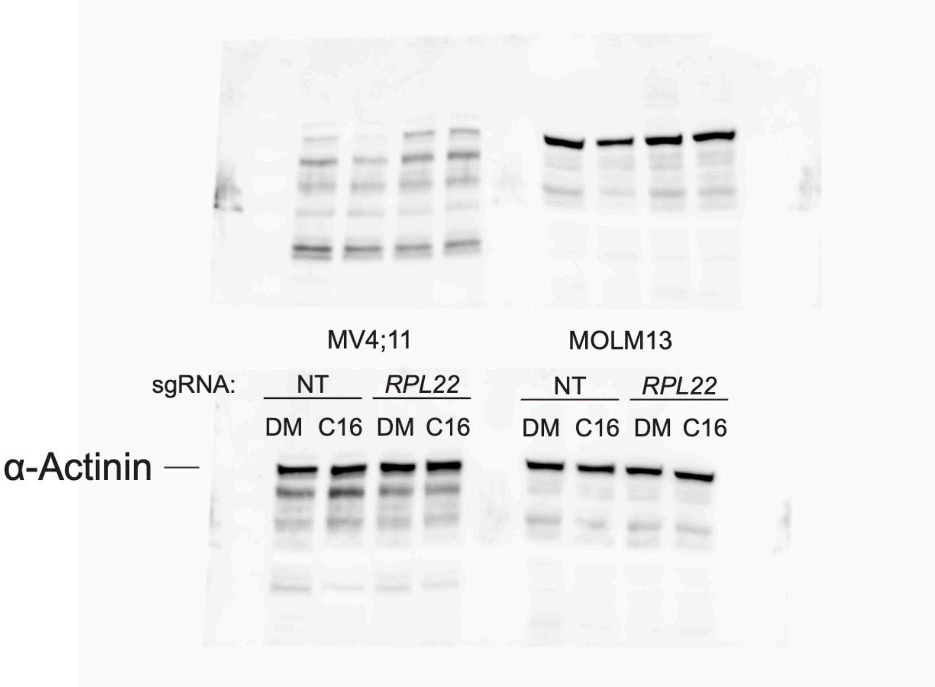

Supplement: Figure 6—source data 5. [file elife-90683-fig6-data5.pdf]
